# Editorial: Insights on neuroinflammatory response by microglia-targeted pharmacology

**DOI:** 10.3389/fphar.2023.1205859

**Published:** 2023-05-04

**Authors:** Jacob Raber, Hector J. Caruncho, Philippe De Deurwaerdere, Massimo Grilli

**Affiliations:** ^1^Behavioral Neuroscience, Oregon Health and Science University, Portland, OR, United States; ^2^ University of Victoria, Victoria, BC, Canada; ^3^ Université de Bordeaux, CNRS UMR5287, Bordeaux, France; ^4^ Department of Pharmacy, University of Genova, Genova, Italy

**Keywords:** microglia, Alzheimer’s disease, Parkinson’s disease, lymphocytes, cancer and cancer treatments

Microglia, the innate immune cells of the CNS, are critical for the regulation of the neuronal network, from the early neuronal developmental stages to adulthood (McGeer and McGeer, 2010). They act as brain macrophages, wiping out cell debris and phagocytizing viruses and bacteria. They support the development, maintenance, homeostasis, and repair of the brain (Eikelenboom and Veerhuis, 1996; Sajja et al., 2016). The resting state of microglia is sensitive to environmental stimuli. For example, stress conditions can activate aberrant microglia functioning, leading to the adverse onset of neurodegenerative and psychiatric disorders. In recent years, morphological ultra-structures and molecular states of microglia related to health and disease conditions have been reported. Recognition of microglia heterogeneity is fundamental to identify microglia-selectively therapies and uncover the underlying mechanisms that activate the reparative and regenerative functions of microglia (Lee et al., 2018). Pro-inflammatory microglia (M1-activated state) secrete proinflammatory cytokines such as tumor necrosis factor-α (TNF-α), interleukin (IL)-1β, IL-6, and inducible nitric oxide synthase (iNOS), which typically lead to dysfunction following chronic activation. In contrast, neuroprotective microglia (M2 state) phagocytose cell debris and misfolded proteins, promote tissue repair and reconstruction of the extracellular matrix and support neuron survival mediated by neurotrophic factors.

**FIGURE 1:**
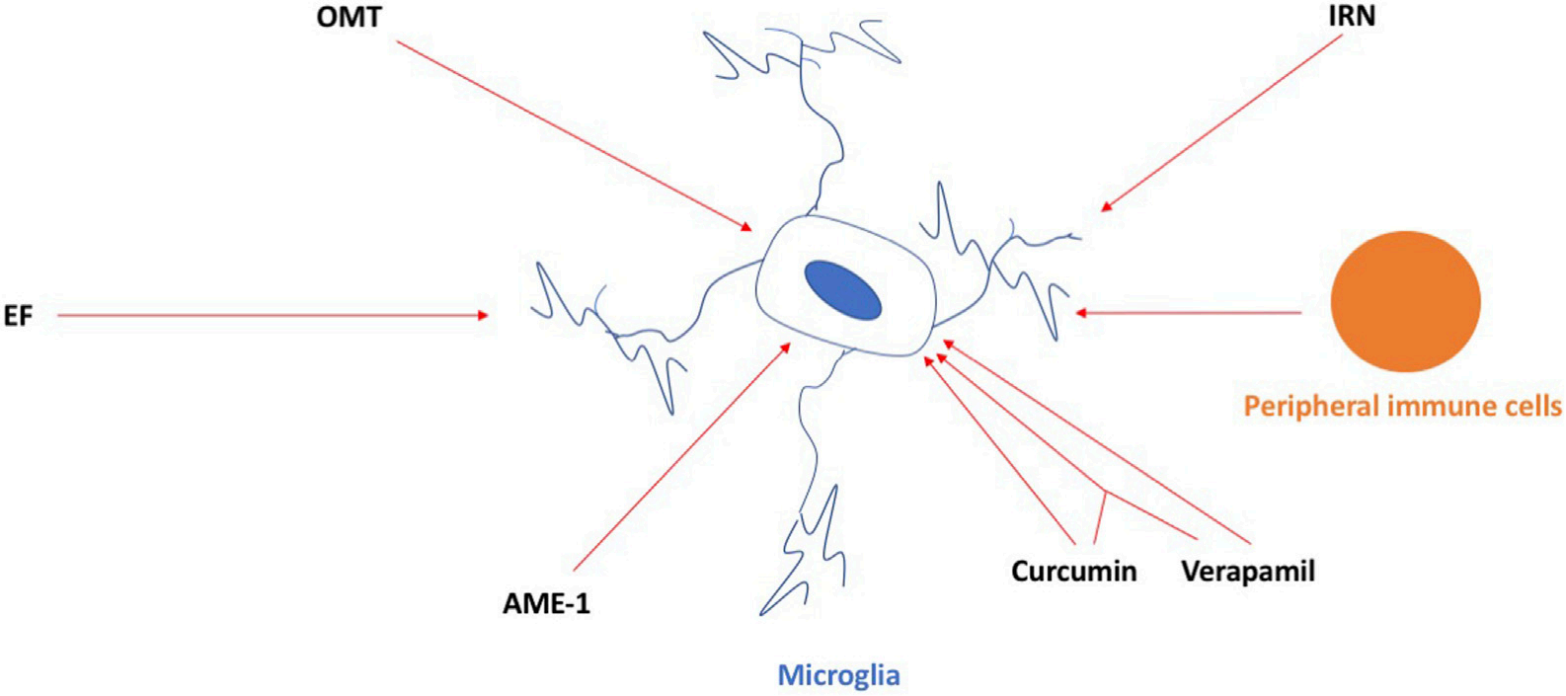
OMT, IRN, EF, AME-1, and curcumin and verapamil, and the combination of both, can inhibit the activation of microglia in neurodegenerative conditions. Peripheral immune cells infiltrating the brain can activate microglia in the brain as well and, in this way, contribute to neuroinflammation in neurodegenerative conditions. For details, see the main text.

In this Research Topic, novel and well-characterized compounds are being discussed as microglia-selective neuro-therapies that can alter the functional state of microglia and modulate neuroinflammation (Figure 1). Gan et al. used oxymatrine (OMT), a natural quinoxaline alkaloid extracted from the root of *Sophora flavescens*, and provided evidence for its ability to reduce neuroinflammation in the 1-methyl-4-phenyl-1, 2, 3, 6-tetrahydropyridine (MPTP) mouse model of Parkinson’s disease (PD). They also used 1-methyl-4-phenylpyridinium (MPP^+^)-induced mice primary microglia, primary murine neuron-microglia co-cultures, and primary microglia infected with cathepsin D (CathD)-overexpressed lentivirus. OMT dose-dependently inhibited MPTP-induced motor deficits and provided dopamine neuroprotection. OMT also inhibited microglia activation following exposure to the MPTP/MPP^+^-induced release of pro-inflammatory cytokines, downregulated the expression of CathD, and inhibited the activation of the HMGB1/TLR4 signaling pathway and the nuclear translocation of NF-κB. Their study supports a potential role for OMT in ameliorating PD and proposes that OMT may be useful in the treatment of PD.

Deng et al. used isorhynchophylline (IRN), a tetracyclic oxindole alkaloid extracted from *Uncaria rhynchophylla* with anti-inflammatory effects in microglial cells, and provided evidence for its ability to provide protection against ischemia/reperfusion injury induced by middle cerebral artery occlusion (MCAO) by employing a rat model of stroke. IRN attenuated the infarct volume, improved the neurological function, and reduced the neuronal death rate, brain water content, and aquaporin-4 expression in the brains. IRN also inhibited IκB-α degradation, NF-κB p65 activation, CX3CR1 expression, microglial activation, and the inflammatory response.

Zou et al. provided evidence for the ability of ethyl ferulate (EF) to improve ischemic stroke outcomes. EF suppressed the lipopolysaccharide (LPS)-induced pro-inflammatory response in the primary microglia and immortalized cell lines and post-stroke neuroinflammation in the transient middle cerebral artery occlusion (tMCAO) stroke model in mice. EF was shown to bind and inhibit the activity of monoamine oxidase B to reduce the pro-inflammatory response.

Wagner et al. used the hallucinogen and mushroom *Amanita muscaria* and provided evidence of the ability of *A. muscaria* extract (AME-1) to modify the inflammatory responses in a human microglia cell line (HMC3) by using flow cytometry. AME-1 upregulated expression of CD86, CXCR4, CD125, and TLR4 receptors. Although AME-1 at higher concentrations increased IL-8 production of HMC3, AME-1 potentiated HMC3 production of IL-8 in response to poly(I:C). Metabolomics analysis revealed that AME-1 contains the autophagy inducer trehalose, which also potentiated the HMC3 poly(I:C)-mediated production of IL-8.

Ortiz-Romero et al. used chronic treatment with the natural phenol compound curcumin, the non-selective voltage-dependent calcium channel verapamil, and a combination of both in a mouse model of the rare neurodevelopmental disorder Williams–Beuren syndrome (WBS) (WBS complete deletion (CD) mice characterized by a 1.3 Mb heterozygous deletion). CD mice have a distinctive cognitive phenotype compared to wild-type, marked by hypersociability and lower performance in the marble burying test. CD mice showed more activated microglia in the motor cortex and CA1 hippocampal region, which were prevented by co-treatment of curcumin and verapamil. Behavioral improvement with the combination of curcumin and verapamil on hypersociability correlated with the molecular recovery of several affected pathways involving MAPK signaling and was important in the control of synaptic transmission and inflammation. The whole study highlighted a possible pharmacological targeting of microglia for a symptomatic treatment of this complex disease that is, currently devoid of treatment.

Finally, McKee et al. contributed a mini-review about microglia as a target in age-related cognitive decline (ACD) and Alzheimer’s disease (AD), discussing microglial pathways of interest for the prevention and treatment of ACD and AD. In addition, they explored the heterogeneity of microglia in these conditions and how pharmacological agents could target specific microglial states. The infiltration of immune cells into the brain might play a role in these detrimental effects of activated microglia. As recently shown by Chen et al. (2023), microglia-mediated T-cell infiltration induces tauopathy, a marker of AD neuropathology, in mice with tauopathy and in AD brains. The number of T cells, especially cytotoxic T cells, was increased in areas with tau pathology and correlated with neuronal loss, and the cells dynamically transformed their cellular characteristics from activated to exhausted states. When tauopathy mice were given a drug or an antibody known to result in the death of microglia or T cells, both decreased brain atrophy. The depletion of microglia reduced the number of T cells in the brain, and T-cell depletion reverted the microglia to a state more like that seen in a healthy brain.

These timely studies and this mini-review support the potential of microglial and infiltrating immune cells as therapeutic targets in neurodegenerative conditions. Future efforts are warranted to further develop and test these interventions in pre-clinical models and subsequently in patients.
